# Assessing Violence Risk among Far-Right Extremists: A New Role for Natural Language Processing

**DOI:** 10.1080/09546553.2023.2236222

**Published:** 2023-07-25

**Authors:** Julia Ebner, Christopher Kavanagh, Harvey Whitehouse

**Affiliations:** Department of Anthropology and Museum Ethnography, University of Oxford, Oxford, UK

**Keywords:** Far-right extremism, violent extremism, terrorism, identity fusion, violence risk assessment

## Abstract

A growing body of research suggests that an individual’s willingness to fight and die for groups is rooted in the fusion of personal and group identities, especially when the group is threatened, violence is condoned, and the group’s enemies are dehumanised or demonised. Here we consider whether the language used by extremists can help with early detection of these risk factors associated with violent extremism. We applied a new fusion-based linguistic violence risk assessment framework to a range of far-right extremist online groups from across the violence spectrum. We conducted an R-based NLP analysis to produce a Violence Risk Index, integrating statistically significant linguistic markers of terrorist manifestos as opposed to non-violent communiqués into one weighted risk assessment score for each group. The language-based violence risk scores for the far-right extremist groups were then compared to those of non-extremist control groups. We complemented our quantitative NLP analysis with qualitative insights that contextualise the violence markers detected in each group. Our results show that the fusion markers combined with several other variables identified across the different online datasets are indeed indicative of the real-world violence level associated with the relevant groups, pointing to new ways of detecting and preventing violent terrorism.

## Introduction

Terrorist attacks from single actor perpetrators aligned with far-right online networks are on the rise.^1^ The 2019 Christchurch attack that killed over fifty people in two consecutive mosque attacks in New Zealand was followed by a series of copycat incidents in other geographies—including the attacks of Halle (Germany), Poway (U.S.), El Paso (U.S.) in 2019, and Buffalo (U.S.) in 2022—seemingly inspired by similar hatreds.^2^ Meanwhile, the past decade was also marked by the growth of internationally organised far-right terrorist networks, such as Atomwaffen Division and National Action, which established regional offshoots and aliases to continue their operations after governmental bans were imposed on them.^3^ The expanding national security threat posed by online far-right extremist networks has highlighted the need for evidence-based risk assessment frameworks that can be applied to cyberspace.

In recent years, an increasing number of studies have focused on identifying potential violence predictors in online spaces and determining promising areas for future research.^4^ Nonetheless, many questions relating to the relationship between online discourse and offline violence remain unresolved. This paper seeks to help fill this gap by exploring potential online predictors of real-world violent extremism based on a new theoretically grounded framework. Beyond its academic contribution, this study could also provide valuable insights for policymakers, social media companies, and frontline practitioners seeking to understand, prevent, and combat online radicalisation towards violence. To better understand how online discourse may be connected to offline violence, it is necessary to combine concepts and methods from multiple disciplines. While the main scholarly contribution of this paper is in the field of terrorism studies, it draws on previous research in social psychology, cognitive anthropology, linguistics, and internet studies.

We argue in this paper that extremist ideologies pose a much greater violent threat when combined with identity fusion, an extreme form of group alignment. Identity fusion (hereafter “fusion” for short) creates a porous boundary between personal and group identities which can serve to motivate extreme forms of pro-group action.^5^ Fused individuals—whose personal and group identities are activated synergistically—have been observed to be more likely to resort to violent forms of self-sacrifice to protect their in-group from a perceived external threat. Studies demonstrating this phenomenon have been carried out in a variety of different contexts, such as among Islamist fundamentalists in Indonesia,^6^ revolutionary battalions in Libya,^7^ herder-farmers in Cameroon,^8^ college fraternities in the U.S.,^9^ and football hooligans in Britain and Brazil.^10^

The analysis presented here uses natural language processing (NLP) methods to analyse the links between online psycholinguistic markers and offline violence. As noted by van der Vegt et al., NLP has brought both opportunities and challenges to threat assessments in the field of counter-terrorism and should be deployed with care.^11^ To address these challenges, our NLP analysis uses a cautious coding approach, which draws heavily on manual reviews as well as Intercoder Reliability (ICR) tests with expert and non-expert coders, as will be outlined in more detail below. Moreover, the quantitative analysis was complemented with qualitative digital ethnographic insights to contextualise the NLP results. In this exploratory study, we test a new linguistic violence risk assessment framework that was informed by our prior linguistic analysis of terrorist manifestos.^12^ The previously conducted comparative manifesto analysis served to trace psycholinguistic markers that were statistically significant in the manifestos of authors who would then commit acts of terrorism in the real world.^13^ To allow for cross-group comparison in this paper we apply the subsequently developed text analysis framework to both extremist and non-extremist groups.

We acknowledge that definitions of extremism, violent extremism, and terrorism radically differ across different geographies, disciplines, and research institutions.^14^ For consistency with our other research projects we apply the *Institute for Strategic Dialogue (ISD)*’s definition of extremism as the advocacy of a system of beliefs “that claims the superiority and dominance of one identity-based ‘in-group’ over all ‘out-groups’” and “advances a dehumanizing ‘othering’ mind-set incompatible with pluralism and universal human rights.”^15^ The ISD’s definition is rooted in Social Identity Theory and draws on JM Berger’s definition of extremism as “the belief that an in-group’s success or survival is inseparable from the need for hostile action against an out-group.”^16^ Violent extremism, as the term suggests, adds a further layer of the willingness to employ violence to advance the beliefs embraced in ideological extremism.^17^ In line with this distinction, our paper differentiates between identity-driven types of extremism, namely ideological fixation on extreme ideologies versus self-sacrificial pro-group violence. In the context of this paper, terrorism-related language will be used to describe violent extremist attacks or groups that meet the legal threshold to be labelled “terrorist.”

As this study specifically explores identity fusion and other psychological metrics in different online contexts, its definition of “group” is rooted in a social psychological understanding of the term.^18^ For the purpose of this paper, a “group” is not defined by the spaces or communication modes through which it operates but rather as a social category for a collection of people that interact, share common goals, and influence each other’s behaviour, attitudes, and beliefs. We will therefore apply the term “online group” both to small, closed groups on encrypted messaging apps (e.g. an alt-right group on Discord or a conspiracy myth group on Telegram) and less formally organised groups on a forum’s discussion board (e.g. a neo-Nazi forum or a football fans’ forum).

## Emerging literature

There has been a growing body of literature seeking to better understand modern-day far-right extremist violence as well as online communities that have served as hotbeds for radicalisation towards anti-government and anti-minority (e.g. antisemitic, anti-migrant, anti-LGBTQ, etc.) violence.^19^ In 2019, Bjorgo and Ravndal conceptualised extreme-right violence, noting that the scholarly focus on jihadist terrorism has led to a significant gap in research that seeks to understand patterns in far-right extremist attacks, their attackers and victims.^20^ Clemmow et al. introduced the overarching concept of lone-actor grievance-fuelled violence (LAGFV), based on their analysis of sixty-eight lone-actor terrorists and 115 solo mass murderers who were shown to occupy a significant shared space.^21^ Research on specific attacks, terrorists and their online environments included studies that analysed the deadly white supremacist Charlottesville rally of 2017,^22^ the Christchurch attack of 2019,^23^ and the expansion of Atomwaffen Division.^24^ Many studies have focused on the alt-right’s vocabulary and subcultural codes, as well as their crossovers with other online communities such as anime, music, and gaming subcultures.^25^ Other related publications have focused on neo-Nazi forums such as Iron March,^26^ and gaming apps hijacked by far-right extremist movements such Discord, Steam and Twitch.^27^

Much of the existing research on far-right extremist terrorism has pointed to ideological pillars such as the “great replacement” or “accelerationism” as sources of inspiration. Far-right extremists—both single actor perpetrators and organised terrorist groups—tend to endorse two intertwined narratives: (1) the so-called “great replacement” myth, which propagates the idea that the existence of white people is threatened by liberal migration policies and rising birth rates among non-white populations,^28^ and (2) the “accelerationist” imperative, which proposes that an imminent race war needs to be accelerated by staging terror attacks.^29^ An increasing number of studies have also examined manipulation and propaganda techniques used by extremist movements to lower the threshold to use physical force. In particular, the gamification of far-right terrorism has attracted much scholarly attention in recent years, as it has been capable of removing psychological inhibitions to violence by blurring the lines between fiction and reality.^30^ Even though these factors may contribute to an environment in which radicalisation towards violence is likely to occur, they do not provide a robust basis for distinguishing between someone who will eventually carry out acts of extreme violence and someone who will cleave to extreme beliefs but not pose a threat to society.

In 2012, Monahan noted that there is little existing evidence of reliable individual risk factors for terrorism and emphasised the need to focus on studying patterns in ideologies, affiliations, grievances, and “moral” emotions.^31^ In the ensuing decade, an increasing number of studies focused on lone actor terrorists and traces of individual risk factors. Gill et al. analysed the sociodemographic networks, ideologies, grievances, and antecedent behaviours of 119 lone-actor terrorists, finding that no uniform profile exists.^32^ However, the study concluded that lone-actor terrorist incidents tended to be preceded by a range of observable activities within the individual’s wider social movement or terrorist group.^33^ Another important empirical study assessed a sample of twenty-two terrorism offenders in Europe between 1980 and 2015 and developed an investigative template for risk of individual terrorism, the Terrorist Radicalization Assessment Protocol (TRAP-18), which outlined eight warning behaviours and ten distal characteristics.^34^

A 2017 systematic review of studies of existing literature found nine variables that had some association with terrorist group membership or engagement in terrorist activities, including age, socioeconomic status, prior arrest, education, employment, relationship status, grievances, geographic locale, and type of geographic area.^35^ Beyond these factors, the study also highlighted the importance of triggering events.^36^ Sarma warned that risk assessments in counter-terrorism tend to have poor predictive value but argued that researchers can make valuable contributions to the field by developing tools that can help assessors process and synthesise information in a structured way.^37^ Most recently, Scrivens analysed the online posts of violent and non-violent extremists on the neo-Nazi forum Stormfront, quantifying the existence of extremist ideologies, personal grievances, and violent extremist mobilisation efforts.^38^ The results showed that the number of ideological and personal grievance posts in the non-violent and comparison groups was higher than in the violent group; the same was true for violent mobilisation posts.^39^ This suggests that neither extremist ideological posts nor online threats and/or announcements of violence are reliable indicators of real-world violence.

Our paper seeks to address gaps in the existing literature by furthering our understanding of linguistically revealed risk factors (e.g. identity fusion). Several decades of research in the fields of social psychology and cognitive anthropology suggest that while identification with an in-group can motivate adherence to extreme beliefs and outgroup hatreds, identity fusion plays a much stronger role in motivating willingness to fight and die for a group.^40^ Identification is derived from sharing identity markers such as group beliefs and practices, whereas identity fusion taps into personal agency in a more visceral way.^41^ For example, traumatizing or deeply transformative events shared with other members of an in-group can help to create the synergistic relationship between self and group that is the hallmark of identity fusion.^42^ This resonates with the above-mentioned finding that triggering events play an important role in pathways to violence. Since fused individuals tend to view members of their in-group as kin-like (e.g. a brotherhood) metaphors of shared blood and kinship language applied to other group members are among the most prominent linguistic indicators of fusion.^43^

Identity fusion alone, however, is not thought to cause radicalisation towards violence. Whitehouse’s 2018 “fusion-plus-threat” model consolidates previous evidence-based findings about the fusion-violence link, arguing that identity fusion can lead to extreme forms of violence on behalf of an in-group when it is combined with other variables such as the perception of an existential threat posed by an out-group.^44^ This study is based on the fusion-plus-threat model’s key hypothesis: When combined with fusion, perceptions of outgroup threat and the belief that only violence can address that threat, provides a “perfect storm” of factors leading to violent extremism. Whereas none of these factors alone necessarily motivates acts of terrorism, it is their unfortunate combination that creates the catalytic potential that counter-terrorist interventions need to recognise and address.

## Approach and methods

Our study combines quantitative and qualitative text analysis to investigate a range of far-right extremist online groups as well as ideologically moderate control groups with a view to assessing the risk that these groups will resort to violence. We apply a new linguistic violence risk assessment framework that is theoretically grounded in the fusion-plus-threat model to the selected groups.^45^ Despite the solid evidence base that underscores the fusion-plus-threat model and its value for violence risk assessment, our study is among the first to apply the model to online contexts.^46^ Our new assessment framework was created based on a preceding comparative linguistic analysis of terrorist manifestos and non-violent manifestos and has been tested in an Intercoder Reliability (ICR) analysis.^47^ Our ICR analysis with two expert coders and twenty-four non-expert coders confirmed the reliability of our coding scheme, resulting in a confidence rate of over 90 percent for most coding categories.^48^ Apart from linguistic markers of identity fusion (e.g. kinship language and metaphors of shared blood), our framework also includes other markers that may occur in combination with fusion, such as existential threat perceptions, demonising, dehumanising, or derogatory language used against the out-group and violence-condoning group norms. More information about the selection of variables associated with our evidence-based risk assessment framework, as well as the metrics we used to measure them, can be found in Appendix 1.

In today’s rapidly changing online environment, where extremist groups are frequently removed or migrate to other platforms, our selection of far-right groups represents a convenience sample. We collected data from different extremist groups based on three criteria: (a) access conditions that are in line with the university’s ethics framework, (b) large enough datasets of at least 1,000 messages to allow for meaningful NLP analysis, and (c) the presence of extremist narratives based on an initial ethnographic assessment that drew on our above-mentioned definition of extremism. Two easily accessible non-extremist control groups were selected for analysis to compare and contrast the results of the far-right extremist datasets. Our aim was to capture a range of public online spaces, including forums, chat rooms, and social media groups. Specifically, data were collected from four online platforms: (1) the Iron March website, which was a violence-endorsing white supremacist discussion forum that was closed in 2017 due to its links to terrorist organisations such as Atomwaffen Division and National Action, (2) the online gaming chat application Discord, which has hosted a variety of ideologically extreme alt-right and conspiracy theory groups, (3) the moderate Mormon discussion platform Third Hour and (4) the football fan forum FootballForums.net, which is a popular non-ideological hobby community that hosts mainly non-violent football fans but may include a small number of hooligans who have resorted to violence in the past. The datasets were scraped and anonymised in accordance with data protection requirements. Table 1 provides an overview of the selected groups, the total message count in the relevant datasets and the timeframes of the chat logs.

**Table 1.** Overview of selected online groups

Group Name and Type of Platform	Group Explanation	Links to Violent Extremism	Cognitive Extremism Spectrum	Number of Scraped Messages	Timeframe of Scraped Messages
Iron March (Banned Discussion Forum)	Iron March was a public web forum used by members of the neo-Nazi terrorist groups Atomwaffen Divison, National Action as well as the Scandinavian far-right extremist Nordic Resistance Movement and the Australian white supremacist Antipodean Resistance. The website was originally created in 2011 by Russian-based nationalist Alisher Mukhitdinov who went by the name Slavros. Its users have been linked to several terror plots and violent extremist incidents. Iron March closed in 2017 and then turned into a searchable database by the anonymous Twitter user @jewishworker in 2019.^a^	Mainly Violent	Ideologically Extreme	1,164	Jul–Nov 2017
Charlottesville 2.0 (Discord)	Charlottesville 2.0 was one of the key Discord servers used by the organisers of the white supremacist “Unite the Right” rally in Charlottesville, Virginia, on August 11–12, 2017. The protest brought together members of the alt-right, neo-Nazi movements, right-wing militias and neo-Confederates. The rally escalated in several instances of violence, including the deadly car attack by James Alex Fields which killed counter-protester Heather Heyer and injured 35 others. The server was active during the timeframe June to August 2017 before it was taken down by Discord. Its content was first leaked by Unicorn Riot.^b^	Partly Violent	Ideologically Extreme	35,607	Jun–Aug 2017
The Anticom Server (Discord)	The Anticom Server was a white supremacist Discord server of the militant group Anti Communist Action. Anticom had overlapping membership with the Atomwaffen division and shared improvised explosives and combat manuals. The Anticom Discord channel was also used by participants of the “Unite the Right” rally in 2017. The server was active during the timeframe February to September 2017. Its content was first leaked by Unicorn Riot.^c^	Partly Violent	Ideologically Extreme	188,302	Feb–Sept 2017
The Right Server (Discord)	The Right Server is a far-right extremist channel on Discord that was founded by former administrators of the extremist pro-Trump Centipede Central chat room. It was active during the timeframe 2017–2021. After The Right Server was deleted by Discord in 2018, a replacement channel continued under the new name The Right Goys, which is Yiddish for non-Jews.^d^ The messages of the original server were first leaked by Unicorn Riot.^e^	Partly Violent	Ideologically Extreme	678,246	Sep 2017–2020
Third Hour (Religious Discussion Forum)	Third Hour is a popular discussion forum for Mormons. The so-called “Forum for Latter-Day Saints” is used as a place for Mormons to discuss topics such as family, marriage and parenting, missionary work, general welfare and conversion stories but also includes gospel boards and advice boards. There are no known links of forum members to violence or extreme interpretations of the Latter Day Saint (LDS) Movement.	Non-violent	Ideologically Moderate	161,977	2004–2020
FootballForums.net (Hobby Discussion Forum)	FootballForums.net is a popular UK discussion forum for football fans. Its users discuss British and international football, including the UEFA Champions League and Europa League, Premier League as well as non-league football and domestic cups. They also have boards for off-topic discussions and more personal conversations. The forum is a mainstream football hobby forum with users who support a range of different football clubs and therefore not expected to include many radical football hooligans.	Mainly non-violent	Non-Ideological	304,910	2000–2020

^a^J. Singer-Emery and R. Bray, “The Iron March Data Dump Provides a Window Into How White Supremacists Communicate and Recruit,” N.Y./Boston: Lawfare, 2020; Upchurch, “The Iron March Forum.”

^b^C. Schiano, “Charlottesville Violence Planned Over Discord Servers: Unicorn Riot Reports,” Unicorn Riot, 2017.

^c^C. Schiano, “Anticom Discord Server Shows Months of Neo-Nazi Incitement,” Unicorn Riot, 2017.

^d^S. Liao, “Discord shuts down more neo-Nazi, alt-right servers,” *The Verge*, February 28, 2018.

^e^J. Alexander, “Discord is purging alt-right, white nationalist and hateful servers,” *Polyglon*, February 28, 2018.

To measure the presence of identity fusion and other relevant violence risk variables in the collected datasets we performed an NLP analysis in R. Our R code was based on a dictionary approach that traced the linguistic markers found to be associated with higher proneness to violence in our comparative manifesto analysis.^49^ The full list of linguistic markers can be found in Appendix 2. We used the grep R function to capture derivatives of relevant terms and phrases (such as different word classes, grammatical forms and spelling mistakes). While this allowed us to minimise the rate of false negatives in our analysis, a very high number of false positives had to be reviewed manually following the computational analysis. We performed sample-based manual reviews of all filtered datasets to eliminate false positives from the quantitative analysis results. A detailed description of our sampling technique can be found in Appendix 3. In addition to our quantitative text analysis we qualitatively assessed the linguistic markers in the context of entire message exchanges in order to provide more in-depth insights into socio-psychological phenomena at play in processes of online radicalisation towards violence. The pre-defined narratives and their associated linguistic markers were used as a guiding framework for the qualitative analysis.

## Quantitative results

The estimated violence risk was determined based on the Violence Risk Index, a weighted score we developed for the purpose of this study, which reflects the statistical relevance of the different narrative categories as measured in our preceding linguistic study of terrorist manifestos (see Appendix 4 for more details).^50^ More specifically, our formula for the Violence Risk Index integrates the weighted average values of three categories of variables: (a) the four statistically highly significant markers (weighted 0.54 in total/0.14 per variable) fusion, out-group dehumanisation, justification of violence and explicit calls to and announcements of violence, (b) the three statistically significant values (weighted 0.25 in total/0.08 per variable) out-group slurs, out-group demonisation and hopelessness of alternative solutions, and (c) the five other relevant variables (weighted 0.21 in total/0.04 per variable) illustrated in the table below that were found to be frequently cited in terrorism literature but were not statistically significant in our analysis of terrorist manifestos.^51^ We decided to include the variables that were shown to be not statistically significant in our analysis (with a significantly lower weighting per variable) to acknowledge their role in previous studies, which suggest that these factors might at least play a marginal role in the violence risk formula. As can be seen in Table 2, the weightings were calculated based on two criteria: statistical significance and number of variables in the respective category. The statistical significance for each narrative category (significance level of *p* < .05) was calculated with the Mann-Whitney U Test (Wilcoxon Rank Sum Test), using the following formula:^52^

$$\begin{array}{rl} & U_{1}=R_{1}-\frac{n_{1}(n_{1}+1)}{2}\\ & \mathrm{or}\\ & U_{2}=R_{2}-\frac{n_{2}(n_{2}+1)}{2} \end{array}$$

**Table 2.** Weight criteria for violence risk index

	Fusion	Dehumanisation	Justification of Violence	Explicit Calls to and Announcements of Violence	Slurs	Demonisation	Hopelessness of Alternative Solutions	Existential Threat	Conspiracy Belief	Inevitable War	Martyrdom Narrative	Violent Role Model
Critical U value	10	10	10	10	10	10	10	10	10	10	10	10
U value	5.5	3.5	3	0	9	9	9	22.5	12	11	10.5	15
**Significance**	very significant (*p* < 0.5) = U values < 6				significant (*p* < 0.05) = U values 6–10			not significant (*p* < 0.05) = U values > 10				
Equal weight distribution	0.08	0.08	0.08	0.08	0.08	0.08	0.08	0.08	0.08	0.08	0.08	0.08
Criteria 1: Number of variables (50 percent)	0.33				0.25			0.42				
Criteria 2: Stat. significance (50 percent)	0.75				0.25			0.00				
**Weight based on Criteria 1 & 2**	**0.54**				**0.25**			**0.21**				

R_1 _= sum of the ranks in sample 1.

R_2 _= sum of the ranks in sample 2.

n_1 _= number of items in sample 1.

Table 3 illustrates the prevalence of the assessed narrative categories and their linguistic markers in the different datasets, as well as providing an estimated violence risk for each group based on the results of the Violence Risk Index. The percentages in the table describe the detected number of messages carrying relevant linguistic markers of each narrative category relative to the overall message count of the relevant data set. While the percentages may seem low, they reflect the prevalence of the specific risk markers found from across the entire dataset in each case, meaning that the comparative proportions between datasets might be considered more informative than the raw percentages.

**Table 3.** Quantitative analysis results

	Statistically highly significant (weighted 0.54)				Statistically less significant (weighted 0.25)			Statistically non-significant values (weighted 0.21)					Violence Risk Value^a^	
	Fusion	Out-Group Dehumanisation	Justification of Violence	Explicit Calls for and Announcements of Violence	Out-Group Demonisation	Out-Group Slurs	Hopelessness of Alternative Solutions	Existential Threat	Belief in Conspiracy of Out-Group	Inevitable War Narrative	Martyrdom Narrative	Violent Role Model	Violence Risk Index	Estimated Violence Risk
**Far-Right Extremist Groups**														
Iron March	0.86 percent	1.03 percent	0.34 percent	1.55 percent	0.95 percent	2.75 percent	0.00 percent	0.43 percent	0.60 percent	0.26 percent	0.00 percent	0.09 percent	**88**	**VERY HIGH**
Charlottesville 2.0	0.42 percent	0.12 percent	0.07 percent	0.25 percent	0.35 percent	2.47 percent	0.01 percent	0.10 percent	0.12 percent	0,01 percent	0,03 percent	0.00 percent	**36**	**HIGH**
Anticom	0.11 percent	0.19 percent	0.03 percent	0.25 percent	0.21 percent	2.10 percent	0.00 percent	0.09 percent	0.05 percent	0.01 percent	0.02 percent	0.00 percent	**28**	**HIGH**
The Right Server	0.17 percent	0.07 percent	0.00 percent	0.10 percent	0.15 percent	1.36 percent	0.00 percent	0.06 percent	0.05 percent	0.00 percent	0.00 percent	0.00 percent	**18**	**MEDIUM**
**Non-Extremist Control Groups**														
Football Forums	0.14 percent	0.00 percent	0.00 percent	0.03 percent	0.14 percent	0.04 percent	0.00 percent	0.00 percent	0.00 percent	0.00 percent	0.00 percent	0.00 percent	**4**	**LOW**
Third Hour	0.06 percent	0.00 percent	0.00 percent	0.00 percent	0.00 percent	0.00 percent	0.00 percent	0.02 percent	0.02 percent	0.01 percent	0.03 percent	0.00 percent	**1**	**LOW**

^a^The following classification scheme was used for the results of the Violence Risk Index: low: < 10, medium: 10–30, high: 30–70 percent, “very high”: > 70.

Our quantitative analysis found that the far-right extremist dataset Iron March has the highest Violence Risk Index, followed by Charlottesville 2.0, Anticom and The Right Server. These scores mirror the groups’ actual ties to violence and terrorist activities in the offline world, providing preliminary evidence that our linguistic socio-psychological assessment framework offers an accurate estimation of the violence risk associated with far-right online groups. Iron March, whose members participated in terrorist and violent extremist activities, scored 88 on our violence risk scale. The groups Charlottesville 2.0 and Anticom had scores between 28 and 36, which reflects the real-world involvement of some of their members in violence at the Charlottesville rally. The Right Server scored 18 on the violence risk scale, which is in line with the group’s lack of known real-world links to terrorism or extreme violence, even though a few members might have engaged in violent hate crimes. For comparison, our analysis of the non-extreme control group datasets yielded the lowest violence risk scores, with Football Forums scoring 4 and Third Hour scoring 1. Football Forums might have hosted individual users who participated in violent hooliganism but these are likely to be isolated cases given the website’s mainstream audience of football fans supporting a range of local and national teams. The popular Mormon forum Third Hour is associated with a clear non-violence stance and there are no known cases of radicalisation towards violence within this religious online community.

## Qualitative results

The aim of our qualitative textual analysis was to complement our quantitative findings with deeper insights into the nature and application of the assessed violence risk variables in online conversations. The following section investigates recurring narrative patterns of fusion and other relevant markers as well as the contexts in which they occurred across the examined datasets. This section provides actual example sentences we detected in our manual review of the anonymised far-right extremist group datasets, which frequently include derogatory and violent language and may therefore be offensive and shocking.

### In-group identity fusion

As mentioned above, kinship terms applied to other members of the in-group are core to linguistic indicators of fusion. Across all far-right extremist groups identity fusion indicators were observed and members frequently called each other “brothers” and “sisters” in their message exchanges. A particularly high rate of fusion markers relative to the overall datasets was identified in the Iron March and Charlottesville 2.0 datasets. Members of the violent neo-Nazi forum Iron March commonly referred to each other as “brothers.” The discussions ahead of the Charlottesville rally revealed anticipation of the “brotherhood” experience that group members associated with white nationalist rallies. One post read, “Anyone else here get hyped every night as the C-ville event gets closer? I’d take going to this rally ANY day over going to the Bahama’s. Nothing can replace the feeling you get at a White Nationalist rally. That feeling of brotherhood and communion is so far beyond anything else that I know.” Members of the Discord channels Anticom and The Right Server also made use of kinship language when talking to or about fellow group members. For example, Anticom users used phrases such as “my brother in faith” and “Come to my defense Anticom brothers.” Likewise, The Right Server members referred to each other as “MAGA brother,” “faith race family,” and “revolutionary brothers.”

Kinship terms were often used to encourage, protect or unify the group. For example, users in the extremist online groups encouraged each other to “help out our white brothers and sisters” and “support our conservative brothers in a defensive manner.” The idea of brotherhood and unity was emphasised by statements such as “F*ck brother wars” and “If there was ever a time where a European brotherhood would be needed, it’s right now.” One user of The Right Server wrote, “I’m an American soldier, an American. Beside my brothers and my sisters I will proudly take a stand, When liberty’s in jeopardy I will always do what’s right, I’m out here on the front lines […].” Some users also called their in-group “family” or “fam” when providing words of encouragement. For example, one user wrote “Keep it up fam. Get jacked so you can look good when you stab commies with a knife,” and another one posted “Peace be upon those who support us anon but when you’re in the real world you need protection, fam.” Instructive and encouraging words that emphasised metaphorical familiar ties included messages such as “Stay hydrated brothers and sisters,” “Just please don’t get killed brother,” “socialize my brothers,” and “HAIL VICTORY BROTHERS.”

Our qualitative analysis revealed that identity fusion was often found in combination with a perceived need to defend the in-group from an existential threat posed by a demonised out-group. Expressions of kinship feelings towards the in-group were often coupled with the idea that it is necessary to defend the group from an outside threat. One user in the Charlottesville 2.0 server wrote, “He who beats down commies with me today is my brother.” Another one commented, “White brothers: battle lines are being drawn whether you like it or not, and you don’t get to choose whether someone else considers you a target/enemy.” Some went as far as to convey the idea of an existential threat to the in-group. Example messages read, “We are out there to imbue meaning into White cultural destruction and awaken our brothers to the reality of what is happening” or “Every little bit counts, we mustn’t let our brothers and sisters be systematically executed in the land that they built!” In some instances, kinship language was used in explicit attempts to strengthen the group cohesion with messages such as “we’re all brothers here” and “no more brother wars.” One user said, “[…] In the end we are brothers in this fight together. We can work out Economics differences after we remove the filth.”

Identity fusion also occurred in tandem with inevitable war narratives and perceived failure of political paths to change: “I know nuclear [war] is coming, but at least we all die as brothers, right?” read one message shared in the Anticom group. Another user noted, “Civics seek to maintain a system that is bent on white genocide. We will not vote our way out of this, but I’m happy to help my white brothers & sisters any time, any place. So I’ll see all you moderates in Cville.” The perceived hopelessness of alternative solutions caused some users to discuss the potential use of violence in the future when the “optics” of the group would no longer be considered relevant: “I agree on both angles. We need a good spearhead that appeals to the public. But yes. If/when the time comes … My NS brothers and I will be the sword. Waiting behind the scenes.”

There was limited evidence of in-group fusion in the non-extremist datasets. A few members in the Football Forums control group referred to each other as “brothers.” One post used kinship language mixed with the Muslim greeting: “Asalaam-u-laikum my Brothers and Sisters. Peace be upon you all. Stay safe and happy.” Members of Third Hour sometimes referred to fellow Mormons as “brothers” and “sisters,” but also used kinship language when talking about people outside of the Mormon community. One user wrote, “I’m not sure if I like the idea of many people from the media bringing their sensibilities into the community of Saints. But they are our brothers and sisters after all. We’ll have to accept them all.”

### Threat to in-group

Perceived external threats to the in-group included existential threat narratives, conspiracy myths about the out-group, and ideas of an inevitable war. Portrayals of an existential threat to the in-group were found across all far-right extremist datasets. “The future of the white race hangs by a nail,” read a message on the Charlottesville 2.0 server. Along similar lines, users in the Anticom server commented, “Patriots are getting destroyed” and “These people want to literally kill us.” Conspiracy myths about the out-group were also present in all extremist groups. The anti-immigrant “great replacement” myth (often referred to as “white genocide”) and the anti-Semitic “Zionist Occupied Government” (ZOG) myth that Jewish elites control all Western governments were most widespread. Narratives of an inevitable race war were mainly observed in the Iron March dataset, reflecting the group’s accelerationist ideologies. “Alt Right is not building a race war, the shitty economy and unemployment is doing that,” posted one user in the forum.

Many messages specifically related the idea of an existential threat to the “great replacement” myth. For example, one Anticom user wrote, “When races mix you destroy the good traits of both races while maintaining the bad ones.” Others commented, “Allowing subversive kike elements to destroy your country” and “I’m more or less witnessing my race being crossbred to extinction right before my eyes. Makes me sick.” A message on the Charlottesville 2.0 server read, “Multiculturalism is just a synonym for white genocide.” Another one said, “It’s not American Genocide, its White Genocide.” Building on “great replacement” ideas, existential threat warnings were often used to call for action to save the white European race. Messages described Europe as being at a crossroads, where it “will literally be destroyed, or their will be massive ring-wing uprisings.” Another post warned that “our Republic will balkanize if we let diversity destroy it.” Threat narratives were often detected in combination with out-group “othering,” as will be outlined below.

Perceived threats to the in-group were found to be rare in the non-extreme control groups. Neither existential threat nor inevitable war narratives were detected in the Football Forums or Third Hour datasets. Several Football Forums users indicated that they believed in conspiracy myths such as the idea that 9/11 was an inside job. However, only few expressed beliefs in more extreme conspiracies. One user admitted believing that “A lot of people are 1000 percent lizards,” including “mostly politicians, but probably a fair few celebrities too.” Likewise, the use of conspiracy myths in Third Hour was rare. A few users appeared to believe that climate change was a hoax.

### “Othering” of out-group

Demonising, dehumanising, and derogatory language was used to describe perceived enemy groups across all far-right extremist datasets. In some instances, sentences combined different forms of out-group “othering.” For example, the message “Kike devils aren’t creative, they just promote our failed experiments” contained dehumanising, derogatory, and demonising language at once. A user in The Right Server wrote “Niggers are coded to commit crimes!” while a message in Iron March read, “Why do [you] think that Fags aren’t a priority? They are a serious threat.” Messages that warned of “satanic thot witches,” “satanist trannies” or “satanic kikes” are other examples of multi-level “othering.”

Demonised out-groups were equated with “cancer,” “Satan” or “witches.” Demonisation of the “globalist elites,” the media and minority groups was particularly widespread. For example, users suggested that liberals were “trying to destroy humanity,” called globalists “cancer,” and described the Pride flag as “the symbol of the UN, of ZOG, of Silicon Valley, of the Democrat Party, of our ethnic and cultural replacement.”^53^ Others blamed “(((Globalist bankers)))” for “DESTROYING AMERICA!”^54^ and viewed the “(((Media)))” as “an agent of the people who are engineering white genocide.” Examples of anti-minority demonisation included sentences such as “Mixed race people exist to destroy the society that made them possible” or “Niggers are literally destroying this country.”

The most common words used in the far-right extremist groups to dehumanise enemy groups or individuals were “filth,” “plague,” “scum,” “animals,” “monkeys,” “rabbits,” “rats,” “dogs,” “reptilians,” “snakes,” “mongrels,” “monsters,” as well as verbs and adjectives associated with animals such as “breed,” “savage,” “feral,” and “infest.” An Iron March member described non-white people as “breeding like rabbits,” while a user on The Right Server wrote, “South African monkeys are literally raping and murdering young innocent children … .” Dehumanisation was observed to be frequently linked to calls for violence against the out-group. Referring to Black people, one user commented, “We should hunt them to extinction, like the animals they are.” Another one wrote, “Honestly, if you breed with blacks you deserve the axe.”

The most frequently used slurs across all datasets were “niggers,” “kikes” and “faggots,” or derivatives of these terms such as “sandnigger,” “kikebook” and “furfag.” We detected a range of other derogatory terms such as “tranny,” “paki” “muzzie,” “spics,” “thots,” “Japs” and others. Example messages included “How far will the kikes let there [sic!] dogs go,” “It’s just incredible how these fags are complete emotional infants” and “My dog hated niggers … like a good boy.” Other users warned of “Asian streetshitters and Spics” and wrote that “Japs are degenerate scum.” “The Jew” (singular, definite article) was also classified as a frequently used derogatory anti-Semitic term due to its association with NS propaganda. For instance, one user on The Right Server commented that “black people are under propaganda from the jew.” Similar to dehumanising terms, derogatory language was often found to be used in conjunction with calls for and glorification of violence.

The non-extremist datasets had a visibly lower occurrence of “othering” language. There were no slurs, demonising or dehumanising phrases detected in the Third Hour dataset. The members of Football Forums occasionally used derogatory words such as “fags” and “spics.” Likewise, there were only isolated cases of out-group demonisation and dehumanisation in Football Forums. One example of dehumanisation was the comparison of politicians and celebrities with lizards. Another post warned of Muslims allegedly wanting to turn “Bradford, Dewsbury, and Tower Hamlets into independent states under Sharia Law” and noted “there’s still a lot of us left, to outbreed yet.”

### Violence-condoning norms

Violence-condoning norms were observed in all examined far-right extremist groups from across the violence spectrum. However, they were less present in The Right Server than in the other three extremist groups. Violence endorsement took the shape of explicit calls for violence and announcements of violence, including death wishes and discussions of acquiring and using specific weapons, as well as justifications of violence, martyrdom narratives, glorification of violent role models and perceived hopelessness of alternative non-violent solutions.

Explicit calls to violence were found in all datasets, except those of the control groups. Posts such as “Gas the kikes. Race war now,” “HANG ALL NIGGERS,” and “Destroy all that does not fit in the Natural order, faggots are right near the top of that list. Rooftop faggots now” are representative examples. There were also announcements of violence by individual group members. For example, users wrote “I wanna kill lefties,” “I’d kill a communist for fun. But for a green card, I’m gonna carve him up real nice,” “I want to shoot the kike,” and “They are going to be massacred when this come to an end.” Sometimes violence announcements occurred in combination with references to martyrdom narratives: “I just want to die on the battlefield so I can go to Valhalla.”

A few users even posted specific plans to use violence. For example, one Anticom user shared his plans to bomb a U.S. government building, writing “HEY GUYS, I HAVE DIAGRAMS OF A MAJOR FEDERAL BUILDING IN THE 517 AREA CODE AND I HAVE BLUEPRINTS FOR GUNPOWDER MANUFACTURE FOR USE IN A LARGE SCALE BOMB, USED CONCURRENTLY WITH FERTILIZER.” Another one detailed the steps for planning an attack with improvised explosives, from “Step 1: look at ISIS kitchen video to make a bomb out of household ingredients” to “Step 4: walk in with backpack and black block and do it Boston bomber style.” Discussions about organising and using weapons such as guns, pistols, assault rifles, and ammunition were visible in the Iron March server, as well as the chat rooms of Charlottesville 2.0 and Anticom.

While the Iron March users were uniformly condoning violence, a stronger internal divide could be observed in the Charlottesville and Anticom servers. Some users shared thoughts to “SHOOT ANTIFA AND THE NATIONAL GUARD,” “Kill traitors and enemies,” and “We are now burning actual faggots.” However, others voiced their disagreement in messages such as “We really need to avoid killing them though” and “we do not bomb, we are a discord of peace.” Reacting to a user who announced that he’d like to “bomb a Pride Parade,” another one responded, “I get that you play Roblox but stop talking about bombing pride parades and assassinations.”

Death wishes and murder fantasies were common on all extremist servers. One user of Anticom wrote “I just want Jews exterminated,” and a user on The Right Server noted, “I’m not racist, but Asians deserve to be exterminated.” Glorification of violence against out-groups was also observed among the members of all extremist groups. “Take a shot every time a nigger gets shot” read a message on The Right Server and a Charlottesville user called on fellow members to “stock pile dead fags now!” Another message said “Anytime people are shooting, bombing, or attacking Jews, a piece of my soul smiles.” Justifications of violence were present in varying degrees across the far-right extremist groups. Members of Iron March justified the use of physical force against perceived enemy groups. One wrote that violence was “what the cause really needs […] Kill.” Another one argued in favour of making use of the military skills acquired by some of the platform’s members to “slay bodies in boot.” Other users suggested that Black people or other minority groups “earned the bullet” or justified the use of violence with the need “to defend your kinfolk from invasion.”

The concept of self-defence was discussed controversially in the Charlottesville and Anticom servers. Responding to a message that called on users to “defend yourselves,” one user wrote, “Defending = attacking antifa if they attack us?” Strategic conversations about the “optics” of proactive violence versus self-defence could be observed among the participants of the Charlottesville rally who were debating which weapons to bring to the rally and under what circumstances to make use of them. “I’ll tell you how to defend yourself: if somebody punches you, do not stab them. You will go to jail,” wrote one user. “Not pacifists, but defenders,” commented another one. After the Charlottesville rally turned violent resulting in the murder of counter-protestor Heather Heyer as well as several other violent confrontations, the reactions in the Charlottesville 2.0 group were mixed. Some members wrote that “no one is defending violence here” and called on others to “blame those responsible for the violence and disassociate from it,” while others continued to be supportive of violence against out-groups.

The non-extremist datasets did not include explicit calls for or announcements of violence, nor did they contain any noteworthy amount of justification of violence narratives, glorification of violent role models, and hopelessness of alternative non-violent solutions. Martyrdom narratives could be observed in the Third Hour discussions, mainly in reference to religious texts. In Football Forums, the few detected mentions of “martyr” and related words occurred in a negative context when members discussed jihadist terrorism.

## Discussion

This paper has described the results of a study showing that linguistic markers associated with proneness to engage in acts of extreme violence can be measured in online groups. The linguistic violence risk markers found across our datasets mirrored the known actual links of the examined groups to real-world violence and terrorist activities, enabling us to test our framework. This exploratory application allows us to develop the Violence Risk Index. We argue this could be used as an initial assessment tool for intelligence agencies as well as tech firms’ policy and intelligence units to provide a basis for their decisions on resource allocation. In practice, this could mean that the index would be applied alongside existing risk assessment frameworks in far-right extremism monitoring units who would then refer groups that have been identified as “high risk” to the relevant teams responsible for launching more in-depth investigations. While this paper focused on group-level analysis only, follow-up studies should seek to validate our findings with more diverse groups and test whether the Violence Risk Index can be used as a basis for the identification at-risk individuals or sub-groups within larger groups.

Our study’s findings highlight the validity of the fusion-plus-threat model. However, they also support alternative approaches to measuring the risk that individuals or groups will resort to violence, such as Kruglanski et al.’s Quest for Significance Model and Bandura’s Moral Disengagement Theory.^55^ Some of the variables analysed in this paper (e.g. out-group demonization, violence-condoning norms, etc.) may be expressed in beliefs associated with far-right ideologies. For instance, the demonisation of outgroups is inherently linked to “the great replacement” myth, while “accelerationist” narratives are marked by violence condoning norms that justify the use of physically forceful action against a pre-defined enemy group. This study contributes an additional analytical layer to existing risk assessment tools and has the potential to complement and improve current predictive policing approaches. While many of the previously performed studies focused on assessing ideologies, grievances, and mobilisation indicators in violent versus non-violent online samples, the Violence Risk Index developed here focuses on subtle psychological indicators that have been consistently linked to violence in group psychology studies.

Our Violence Risk Index is based on a comparative analysis of manifestos written by terrorists as opposed to manifestos by non-violent (ideologically extreme and non-extreme) authors. This might prompt the question whether our text analysis framework can be applied to more informal, ad-hoc forms of communication in online groups. Given that many of the socio-psychological linguistic markers used in our framework operate on a subconscious level and are not influenced by strategic wording choices, we argue that they can be observed in both formal and informal forms of communication. However, future research should explore the differences in the psycholinguistic patterns of published manifestos as opposed to casual online forum conversations.

The datasets analysed in this paper do not offer a representative account of the multitude of far-right extremist communities found in cyberspace. Our aim was to cover a variety of far-right extremist groups that differ in terms of their actual links to violence rather than offer a comprehensive perspective on the phenomenon. Moreover, we acknowledge that the nature of our selected groups varies in terms of online infrastructure and timeframe of communication, which might impact the analysis results. Access and ethics constraints prevented us from using data from closed chatgroups that have not previously been published and made it difficult to catch groups that operated in exactly the same timeframe. This means that the far-right extremist datasets date back between two and five years and can no longer be found online due to content removal policies. Additionally, we recognise that our sample decisions limit the conclusions that can be drawn. There are also a number of outstanding questions, including whether there are groups that would score high on our index without being associated with real world violence, and whether there are comparable trends in jihadist groups, left-wing extremist groups or in groups that emphasise interpersonal kinship and physical competition but are usually not violent (for instance competitive martial arts teams). These are issues that need to be investigated in further depth before any strong conclusions about the relationship with violence can be validated. Future studies could complement the text analysis of historic datasets with live ethnographic research in extremist online environments.

Our study is explorative in nature and seeks to lay the groundwork for future research. The linguistic markers and R code we used for our NLP analysis can be further developed and expanded. Future research should also perform statistical analysis for violence risk markers observed across different datasets. While our analysis provides a comparative assessment based on the newly developed Violence Risk Index, which is based on statistical analysis of terrorist manifestos, the power and quality of inference of this study are impacted by the absence of statistical comparison of the results for the different groups. Follow-up projects could examine a larger number of groups and statistically compare resulting scores.

Another limitation stems from the psychological and often metaphorical nature of the analysed narrative categories and linguistic markers. Many linguistic markers of our narrative categories inevitably yielded a very high rate of false positives. For example, conversations about real animals were captured in the dehumanisation datasets and chats about actual family members were included in the identity fusion datasets. This meant that the initial R-filtered datasets had to be reviewed manually to ensure only relevant markers would feature in the final datasets. The need for manual reviews was further increased due to our use of the R grep function, as described above, to minimise false negatives and capture derivations of keywords. Our manual reviews to remove false positives from our NLP analysis results were informed by our coding framework, which was previously tested in an Intercoder Reliability (ICR) analysis with the help of two expert coders and twenty-four non-expert coders. However, the messages in our manual sample reviews were often ambiguous and subject to interpretation. For example, “plague” could be read as either demonisation or dehumanisation, depending on the context. The sentence “I am fighting for what I believe is right, not dreaming of some goofy revolution” could be interpreted as a physical or a metaphorical fight. We sought to address these challenges in our qualitative assessment that explored the nature and context of messages in more depth. Finally, it is important to emphasise that while the Violence Risk Index can be used as an evidence-based aid to assess which groups deserve more attention from intelligence agencies than others, it cannot replace human investigations and will need to be combined with manual reviews.

Despite these limitations and the need for future research, as indicated above, the approach adopted here promises to provide a more reliable method of addressing the needle-in-a-haystack problem posed by extremist movements whose members are mostly harmless but among whom future perpetrators of violence lurk. Our goal is to help identify at-risk online groups and individuals in the crowd before they act, using the tell-tale traces they leave behind in their writings. Our research suggests that a lethal cocktail of psychological factors giving rise to violent extremism is fusion and threat combined with various forms of outgroup hatred and violence condoning norms—all of which are detectable in language used in online communications. Understanding the significance of these linguistic markers may hold the key to predicting acts of violent extremism and implementing preventative measures before it is too late.

## Supplementary Material

Supplemental Material

## Data Availability

Due to the sensitivity of the analysed content, the researchers refrain from publishing any raw datasets or evidence screenshots. However, all data can be made available upon request to academics and experts who can provide proof of their affiliation with an independent research institution.

## Notes

1. E. Assoudeh and L. Weinberg, “In Western Europe, Right Wing Terrorism is on the Rise,” Open Democracy, 2021; R. Coolsaet and T. Renard, “What the Zeitgeist can Tell us About the Future of Terrorism,” ICCT Policy Brief (The Hague: International Centre for Counter Terrorism, 2022).

2. J. Ware, “Testament to Murder: The Violent Far-Right’s Increasing Use of Terrorist Manifestos,” ICCT Policy Brief (The Hague: International Centre for Counter Terrorism, 2020); J. Kupper, T. K. Christensen, D. Wing, M. Hurt, M, Schumacher, and R. Meloy, “The Contagion and Copycat Effect in Transnational Far-Right Terrorism: An Analysis of Language Evidence,” *Perspectives on Terrorism* 1, no. 4 (2022): 4–26.

3. A. A. Mattheis, “Atomwaffen Division & Its Affiliates on Telegram: Variations, Practices, & Interconnections,” Research Brief, RESOLVE Network, 2022; Chris Allen, *National Action: Links Between the Far Right, Extremism and Terrorism* (Leicester: UK Commission for Countering Extremism, 2019).

4. See for example K. Cohen, F. Johansson, L. Kaati, and J. C. Mork, “Detecting Linguistic Markers for Radical Violence in Social Media,” *Terrorism and Political Violence* 26, no. 1 (2014): 246–56; L. Kaati, A. Shrestha, and K. Cohen, “Linguistic Analysis of Lone Offender Manifestos” (Proceedings of the 2016 IEEE International Conference on Cybercrime and Computer Forensics, Vancouver); R. Scrivens, P. Gill, and M. Conway, “The Role of the Internet in Facilitating Violent Extremism and Terrorism: Suggestions for Progressing Research,” in *The Palgrave Handbook of International Cybercrime and Cyberdeviance*, ed. T. J. Holt and A. Bossler (London: Palgrave, 2020), 1–22.

5. W. B. Swann, A. Gómez, D. Seyle, J. F. Morales, and C. Huici, “Identity Fusion: The Interplay of Personal and Social Identities in Extreme Group Behavior,” *Journal of Personality and Social Psychology* 96 (2009): 995–1011; H. Whitehouse, “Dying for the Group: Towards a General Theory of Extreme Self-Sacrifice,” *Behavioral and Brain Sciences* 41 (2018): 1–62.

6. C. Kavanagh, S. Wibisono, R. Kapitány, W. Yustisia, I. Eka Putra, A. Rufaedah, and H. Whitehouse, “Exploring the Role of Identity Fusion and Group Identification in Predicting Parochialism amongst Indonesian Islamic groups,” *PsyArXiv* (2019).

7. H. Whitehouse, B. McQuinn, M. Buhrmester, and W. B. Swann, “Brothers in Arms: Warriors Bond like Family,” *Proceedings of the National Academy of Sciences* 111, no. 50 (2014): 17, 783–5.

8. M. D. Buhrmeister, D. Zeitlyn, and H. Whitehouse, “Ritual, Fusion, and Conflict: The Roots of Agro-Pastoral Violence in Rural Cameroon,” *Group Processes & Intergroup Relations* 25, no. 1 (2020): 298–311.

9. H. Whitehouse, J. Jong, M. D. Buhrmeister, A. Gómez, C. M. Kavanagh, B. Bastian, M. Newson, M. Matthews, J. A. Lanman, R. McKay, and S. Gavrilets, “The Evolution of Extreme Cooperation Via Shared Dysphoric Experiences,” *Scientific Reports* 7 (2017): 1–10.

10. M. Newson, “Football, Fan Violence, and Identity Fusion,” *International Review for the Sociology of Sport* 54, no. 4 (2019): 431–44.

11. I. Van der Vegt, B. Kleinberg, and P. Gill, “Proceed with Caution: On the Use of Computational Linguistics in Threat Assessment,” *Journal of Policing, Intelligence and Counter Terrorism* 18, no. 2 (2023): 231–39.

12. J. Ebner, C. Kavanagh, and H. Whitehouse, “Is There a Language of Terrorists? A Comparative Manifesto Analysis,” *Studies in Conflict and Terrorism*, advance online publication. https://www.tandfonline.com/doi/full/10.1080/1057610X.2022.2109244

13. Ibid.

14. D. Koehler, “Violence and Terrorism from the FarRight: Policy Options to Counter an Elusive Threat,” ICCT Policy Brief (The Hague: International Center for Counter Terrorism, 2019).

15. See Institute for Strategic Dialogue, “Extremism,” 2022, Online: https://www.isdglobal.org/extremism/.

16. J. M. Berger, *Extremism. The MIT Essential Knowledge Series* (Cambridge, MA: The MIT Press, 2018).

17. T. Bjørgo and J. A. Ravndal, “Extreme-Right Violence and Terrorism: Concepts, Patterns, and Responses,” ICCT Policy Brief (The Hague: International Centre for Counter Terrorism, 2019).

18. Whitehouse, “Dying for the Group”; H. Whitehouse and J. A. Lanman, “The Ties That Bind Us: Ritual, Fusion, and Identification,” *Current Anthropology* 55, no. 6 (2014): 674–95.

19. L. Munn, “Alt-Right Pipeline: Individual Journeys to Extremism Online,” *First Monday* 24, no. 6 (2019), https://firstmonday.org/ojs/index.php/fm/article/view/10108/7920; A. Klein, “From Twitter to Charlottesville: Analyzing the Fighting Words Between the Alt-Right and Antifa,” *International Journal of Communication* 13 (2019); I. Van der Vegt, M. Mozes, P. Gill, and B. Kleinberg, “Online Influence, Offline Violence: Language use on YouTube Surrounding the ‘Unite the Right’ Rally,” *Journal of Computational Social Science* 4 (2020): 333–54; J. Guhl, J. Ebner, and J. Rau, *The Online Ecosystem of the German Far-Right* (Berlin/London: Institute for Strategic Dialogue, 2018).

20. Bjørgo and Ravndal, “Extreme-Right Violence and Terrorism.”

21. C. Clemmow, P. Gill, N. Bouhana, J. Silver, and J. Horgan, “Disaggregating Lone-Actor Grievance-Fueled Violence: Comparing Lone-Actor Terrorists and Mass Murderers,” *Terrorism and Political Violence* 34, no. 3 (2020): 558–84.

22. J. Davey and J. Ebner, *The Fringe Insurgency: Connectivity, Convergence and Mainstreaming of the Extreme Right* (London: Institute for Strategic Dialogue, 2017); M. A. Peters and Tina Besley, “White Supremacism: The Tragedy of Charlottesville,” *Educational Philosophy and Theory* 49, no. 14 (2017): 1309–12; E. Blout and P. Burkart, “White Supremacist Terrorism in Charlottesville: Reconstructing ‘Unite the Right,’” *Studies in Conflict & Terrorism*, advance online publication. doi: 10.1080/1057610X.2020.1862850

23. J. Davey and J. Ebner, *The Great Replacement: The Violent Consequences of Mainstreamed Extremism* (London: ISD, 2019); S. Lakhani, *The Gamification of Violent Extremism: An Empirical Exploration of the Christchurch Attack* (Washington, DC: Global Network on Extremism & Technology, 2022); G. Macklin, “The Christchurch Attacks: Livestream Terror in the Viral Video Age,” *Westpoint: CTC Senitel* 12, no. 6 (2019): 18–27.

24. J. Ware, “Siege: The Atomwaffen Division and Rising Far-Right Terrorism in the United States,” ICCT Policy Brief (The Hague: International Centre for Counter Terrorism, 2019).

25. M. Fielitz and H. Marcks, *Digital Fascism: Challenges for the Open Society in Times of Social Media* (Berkely: Institute for Peace Research and Security Policy at the University of Hamburg and Berkeley Center for Right-Wing Studies Working Paper Series, 2019); M. Dafaure, “The ‘Great Meme War:’ The Alt-Right and its Multifarious Enemies,” *Angles* 10 (2020): 1–28.

26. H. E. Upchurch, “The Iron March Forum and the Evolution of the ‘Skull Mask’ Neo-Fascist Network,” *West Point: CTC Senitel* 14, no. 10 (2021): 27–37.

27. J. Davey, “Gamers Who Hate: An Introduction to ISD’s Gaming and Extremism Series,” Policy Brief (London: Institute for Strategic Dialogue, 2021).

28. Davey and Ebner, *The Great Replacement*.

29. M. Dittrich, J. Rathje, T. Manemann, and F. Müller, *Militant Accelerationism Origins and Developments in Germany* (Berlin: Center for Monitoring and Analysis (CeMAS), 2022).

30. J. Ebner, *Going Dark: The Secret Social Lives of Extremists* (London: Bloomsbury, 2019); G. Lamphere-Englund and J. White, *The Buffalo Attack and the Gamification of Violence* (London: RUSI, 2022); L. Schlegel, “Connecting, Competing, and Trolling: ‘User Types’ in Digital Gamified Radicalization Processes,” *Perspectives on Terrorism* 15, no. 4 (2021): 54–63.

31. J. Monahan, “The Individual Risk Assessment of Terrorism,” *Psychology, Public Policy, and Law* 18, no. 2 (2012): 167–205.

32. P. Gill, J. Horgan, and P. Deckert, “Bombing Alone: Tracing the Motivations and Antecedent Behaviors of Terrorists,” *Journal of Forensic Sciences* 59, no. 2 (2014): 425–35.

33. Ibid.

34. J. R. Meloy, K. Roshdi, J. Glaz-Ocik, and J. Hoffmann, “Investigating the Individual Terrorist in Europe,” *Journal of Threat Assessment and Management* 2, no. 3–4 (2015): 140–52.

35. S. L. Desmarais, J. Simons-Rudolph, C. S. Brugh, E. Schilling, and C. Hoggan, “The State of Scientific Knowledge Regarding Factors Associated with Terrorism,” *Journal of Threat Assessment and Management* 4, no. 4 (2017): 180–209.

36. Ibid.

37. K. M. Sarma, “Risk Assessment and the Prevention of Radicalization from Nonviolence into Terrorism,” *American Psychologist* 72, no. 3 (2017): 278–88.

38. R. Scrivens, “Examining Online Indicators of Extremism among Violent and Non-Violent Right-Wing Extremists,” *Terrorism and Political Violence*, advance online publication. https://www.tandfonline.com/doi/full/10.1080/09546553.2022.2042270

39. Ibid.

40. Whitehouse and Lanman, “The Ties That Bind Us”; Whitehouse et al., “The Evolution of Extreme Cooperation,” 1–10.

41. Whitehouse, “Dying for the Group.”

42. Whitehouse et al., “The Evolution of Extreme Cooperation.”

43. Whitehouse and Lanman, “The Ties That Bind Us.”

44. See note 41 above.

45. Ibid.

46. Ibid.

47. Ebner et al., “Is There a Language of Terrorists?”

48. Ibid.

49. Ibid.

50. Ibid.

51. The following formula was used for our Violence Risk Index: Average value (Stat high variables)*0.54 + Average value (Stat low variables)*0.25+ Average (Stat insign variables)*0.21. The results were multiplied by 100 for improved readability.

52. To be statistically significant, the smaller of the two U-values (U_1_ and U_2_) has to be equal to or less than the critical U-value (See Appendix 4).

53. ZOG stands for “Zionist Occupied Government” in reference to the anti-Semitic conspiracy myth that Jews are controlling all Western governments.

54. Triple brackets are an anti-Semitic symbol used by the alt-right to signal that someone or something is supposedly Jewish.

55. See A. Kruglanski, K. Jasko, and D Webber, “The Making of Violent Extremists,” *Review of General Psychology* 22, no. 1 (2018): 107–120; A. Bandura, “The Origins and Consequences of Moral Disengagement: A Social Learning Perspective,” in *Understanding Terrorism: Psychosocial Roots, Consequences, and Interventions*, ed. F. Moghaddam and A. J. Marsella (Washington, DC: American Psychological Association, 2004).

