# Supplementary material for: Assessing Violence Risk among Far-Right Extremists: A New Role for Natural Language Processing
Source: Terror Political Violence. 2023 Jul 25;36(7):944–61. doi: 10.1080/09546553.2023.2236222 (PMC11382783; doi:10.1080/09546553.2023.2236222)
Supplement: Supplemental Material [file FTPV_A_2236222_SM5512.docx]

**Appendices**

*Please note that parts of the appendices have been published in other peer-reviewed articles.*

**Appendix 1: Relevant Variables and Definitions**

For this study we selected variables that were previously identified as potential mediators or moderators of the fusion to violence pathway. Of particular note due to being frequently cited factors that might contribute to an escalation towards violence include:

1. Perceived out-group entitativity,^[[1]](#endnote-1)^ which will be traced via the use of “us versus them” narratives^[[2]](#endnote-2)^, in particular the use of language that insults, demonizes or dehumanizes an entire out-group,^[[3]](#endnote-3)^
2. perceived out-group threat,^[[4]](#endnote-4)^ which may manifest itself in narratives of an existential threat posed to the in-group,^[[5]](#endnote-5)^ the belief in a conspiracy of the outgroup,^[[6]](#endnote-6)^ or the belief in an inevitable war between the in- and out-group,^[[7]](#endnote-7)^ and
3. violence condoning norms,^[[8]](#endnote-8)^ which may include the justification of violence, the glorification of violence via martyrdom narratives or the so-called “warrior mentality”,^[[9]](#endnote-9)^ the identification with a violent role model,^[[10]](#endnote-10)^ and perceived hopelessness of alternative solutions.^[[11]](#endnote-11)^

Figure 1. Proposed Relationships Between Group Alignment and Behavioural Outcome


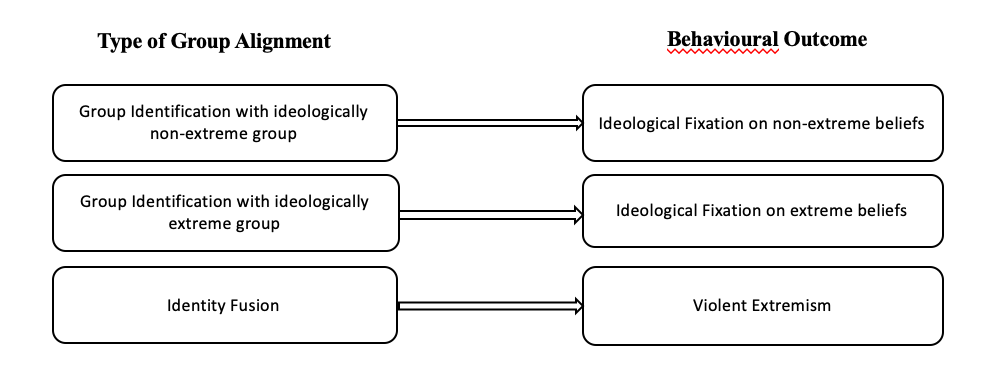


Figure 2. Proposed Relationship Between Fusion and Violence

#
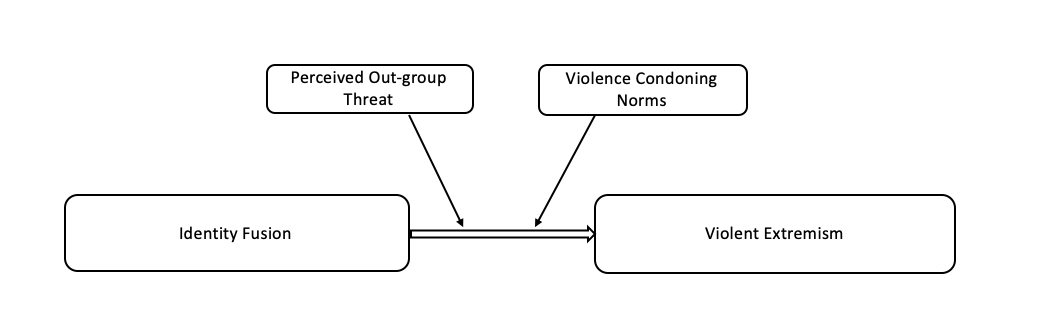


| **Meta -Category** | **Sub-Category** | **Definition** |
| --- | --- | --- |
| **Group alignment** | **In-Group Identification** | *In-Group Identification* describes an individual’s sense of belonging to a defined group in social psychology (Pennebaker and Chung 2008). Previous studies found that in-group identification can be reflected in the use of first-person plural pronouns such as “we”, “us”, “our”.^[[12]](#endnote-12)^ |
|  | **In-Group Identity Fusion** | *In-Group Identity Fusion* is a socio-psychological concept that describes a process where an individual’s identity merges with the group identity. This dynamic is usually characterized by the use metaphors of kinship and family relatedness when talking about the in-group: e.g. words such as "brother", "sister", "loyalty" “family” “sons” “daughters” “our blood” “brotherhood” “motherland” “fatherland” might be used to talk about the in-group and/or fellow group members.^[[13]](#endnote-13)^ |
| **Out-Group Entitativity** | **Out-Group Slurs** | *Out-Group Slurs* are derogatory terms used in the context of hate speech and extremist texts.^[[14]](#endnote-14)^ They are offensive labels used to describe an entire group of people based on their ethnicity, race, gender, religion or sexuality.^[[15]](#endnote-15)^ Well known examples are “kike”, “kufar”, “infidel”, “fag”, “negro”, “spic”, “the Jew”, the n-word or similar terms.^[[16]](#endnote-16)^ |
|  | **Out-Group Demonization** | *Out-Group Demonization* describes “the attribution of basic destructive qualities to the other“,^[[17]](#endnote-17)^ or the blaming of the out-group for the personal misfortunes or the in-group.^[[18]](#endnote-18)^ It usually involves the framing of an out-group as bad, hostile or threatening to the in-group. For example, studies explain that depictions of Jews as the “devil”, “sly conspirators”, “greedy Shylocks” or “vengeful beneficiaries” have been used to demonize them as a dangerous out-group.^[[19]](#endnote-19)^ |
|  | **Out-Group Dehumanization** | *Out-Group Dehumanization* “involves viewing others as less than human”, for example by describing them as or comparing them with animals.^[[20]](#endnote-20)^ Beyond the literal comparison with animals such as “monkey”, “donkey, “dog”, non-human related words applied to members of an out-group such as “creature”, “tame” and “breed” could also be indicative of out-group demunisation.^[[21]](#endnote-21)^ |
| **Out-Group Threat** | **Existential Threat to In-Group** | *Existential Threat to In-Group* summarizes the idea of the in-group being threatened with physical or symbolic collective annihilation.^[[22]](#endnote-22)^ This might express itself in the belief that the in-group is facing a genocide or coordinated attack: for instance, some far-right extremist groups argue that white populations are facing an existential threat because they are dying out demographically due to immigration, abortion, and violence against whites.^[[23]](#endnote-23)^ |
|  | **Belief in Out-Group Conspiracy** | *Belief in Out-Group Conspiracy* denotes a functionally integrated mental system which assumes that “a group of actors collude in secret to reach malevolent goals”.^[[24]](#endnote-24)^  A linguistic analysis of the subreddit  r/conspiracy found that compared to the control group the conspiracy theory community made more frequent use of words related to the categories “crime”, “stealing” and “law”.^[[25]](#endnote-25)^ |
|  | **Belief in Inevitable War** | *Belief in Inevitable War* involves the idea that a war of races, religions, cultures or other opposing groups is looming above the in-group and cannot be prevented, or that a war between the in- and out-group is already under way. Inevitable war narratives are closely linked to “Accelerationism”, which describes the desire to trigger a looming and inevitable violent escalation of existing tensions and societal collapse.^[[26]](#endnote-26)^ |
| **Violence Condoning Norms** | **Justification of Violence** | *Justification of Violence* include rational or emotional reasonings of why resorting to violence is the best or only solution.^[[27]](#endnote-27)^ For example, research highlighted group norms within jihadist groups that suggested a moral justification of terrorism and violent action via the ideas of pre-emptive action, self-defence or escape from a deleterious condition that requires an immediate action.^[[28]](#endnote-28)^ |
|  | **Martyrdom Narrative** | *Martyrdom Narrative* describes the glorification of violence and terrorism by framing past or future violent action by in-group members against the out-group as heroic, selfless acts that serve a bigger purpose. For example, the language and symbolism of martyrdom might appear in the form of references to “heroic martyrs”, “resistance”, “self-sacrifice” or “dying in glory”.^[[29]](#endnote-29)^ |
|  | **Violent Role Model** | *Violent Role Models* may be mentioned in manifestos by invoking well-known perpetrators of genocidal violence as sources of inspiration**.^[[30]](#endnote-30)^** For example, authors might indicate support of previously successful terrorists by expressing identification, support or admiration (e.g. “I admire”, “I salute”, “I support”, naming someone “Saint”, “God”, etc.) for previous terrorists.^[[31]](#endnote-31)^ |
|  | **Hopelessness of Alternative Solutions** | *Hopelessness of Alternative Solutions* summarizes the perceived failure of non-violent solutions such as political, diplomatic or other peaceful activist means**.** Authors of manifestos may indicate that they have “nothing to lose” or that “democracy/politics have failed” and therefore resort to more extreme solutions.^[[32]](#endnote-32)^ |
| **Violence Threats** | **Calls to Violence** | Calls to Violence cover announcements of violence and/or extreme self-sacrifice committed by the author as well as calls that encourage the manifesto’s readers to engage in violence and/or self-sacrifice against a defined out-group. Words such as “kill”, “shoot”, “hang”, “bomb”, “slaughter” or “assassinate” may be indicative but calls to violence may also reference specific weapons such as “sniper rifles”, “ammonium nitrate”, etc.^[[33]](#endnote-33)^ |

**Appendix 2: Linguistic Markers used in NLP analysis**

|  | **Narrative** | **Detected Keywords** |
| --- | --- | --- |
| **Group Alignment** | **In-Group Identification** | *“We”/”Us”/”Our” in combination with* “European”, “cultural conservative”, “Christian conservative”, “conservative”, “indigenous”, “non-Muslim”, “Justiciar Knight”, “patriot”, “martyr”, “nationalist”, “my people”, “my race”, “our race”, “anon”, “white men”, “whites”, “Aryan”, “true Muslim”, “believer”, “Muslim community”, “ummah”, “Muslim society” |
|  | **In-Group Identity Fusion** | “brother”, “sister”, “sons”, “daughters”, “kin”, solidarity”, “family”, “fellow …”, “comrades”, “my blood”, “our blood”, “bloodline”, “ancestry”, “descendant”, “ancestor”, “brethren” (These terms only indicate identity fusion when used metaphorically to describe the in-group rather than biological family.) |
| **Violence Threats** | **Calls to Violence** | “executed”, “execution”, “punished”, “punishment”, “death penalty”, “kill”, “massacre”, “attack”, “destroy”, “retribution”, “revenge”, “punish”, “eradicate”, “starve”, “die”, “torture”, “behead”, “guns”, “must attack”, “must fight”, “must kill”, “give them hell”, “must play his part in this revolution”, “burn”, “shoot”, “flamethrowers”, “firearm”, “weapon”, “grenade”, “bomb”, “set fire”, “Molotov”, **“**fight”, “brutal steps”, “jihaad”, “bring death to”, “forcible overthrow”, “revolution” |
| **Out-Group Entitativity** | **Out-Group Slurs** | “kike”, “nigger”, “negro”, “spic”, “fag”, “goyim”, “golem”, *“the Jew”, “global Jewry”, “pajeet”, “bitch”, “whore”* |
|  | **Out-Group Demonization** | “traitor”, “corrupt”, “evil”, “enemy”, “our enemies”, “vicious”, “barbaric”, depraved”, “vile”, “puppets”, “perversion”, “blood libel” “crimes”, “cruel”, bloody”, genocidal”, “sinful”, deceitful”, “invader”, “poison”, parasite, “menace”, “brutal”, “ruthless”, “ “bloodsucking”, “dirty”, “deceptive”, “treacherous” “poisonous”, “oppression” , “oppressive”, “shirk” “unbeliever”, “immoral” “jahili”, “pollute”, “demolish”, “shake the foundations”, “Dar- ul-Harb”, “arrogant”, “mischievous”, “criminal”, “deceivers”, “liars” |
|  | **Out-Group Dehumanization** | “animal”, “plague”, “impure”, “brute”, “dog”, “lower Iq”, “lower being”, “inferior”, “squalid” “parasitic”, “parasite”, “creature”, “trash”, “filth”, “vermin”, “spider”, “devil”, “monster”, “beast”, “reptile”, “reptilian”, “snake”, “cockroach”, “beneath human skin”, “scum” |
| **Out-Group Threat**  10. | **Existential Threat to In-Group** | “subjected to”, “coerced”, “brainwashed”, “exterminated”, “brutalised”, “raped”, “terrorised”, “ravaged”, “robbed”, “replace”, “subjugate”, “make war upon my people” “destroyed”, “overwhelmed”, “under siege”, “under demographical siege”, “disenfranchise”, “subvert”, “destroy”, “assault”, “kill us”, “kill our…”, “running out of time”, last chance”, “enslavement”, “suffer”, “economic plunder”, “condemned to death”, destruction of all mankind”, “ill society”, “at the brink of”, “danger”, “annihilation”, “extinction”, “decay” |
|  | **Belief in Out-Group Conspiracy** | “betray”, “betrayal”, “sell”, “sold”, “collude against”, “colluded”, “conspire”, “fake”, “fraud”, “corruption”, “corrupt”, “ZOG”, “Kalergi”, “white genocide”, “great replacement” |
|  | **Belief in Inevitable War** | “war”, “battle”, “fight”, “jihaad” *in combination with* “imminent”, “inevitable”, “looming”, “started”, “already” |
| **Violence Condoning Norms**  14. | **Justification for Violence** | “pre-emptive”, “defend”, “protect”, “self-defense”*,* “self-defence”, “forced to fight”, “no longer ignore”, “act of defense”, “purified”, “purify”, “brutal steps should have been used”, “need for jihaad”, “reasons for jihaad”, “need for war”, “the struggle is imposed upon”, “natural struggle”, “cannot co-exist” |
|  | **Martyrdom Narrative** | “die in glory”, “sacrifice”, “knight”, “martyr”, “dying selflessly”, protecting our people”, “immortal”, “act of preservation”, “my death”, “defending the work of the Lord”, “standing guard”, “appears as the herald”, “release mankind from servitude“, “free from”, “freed from” |
|  | **Violent Role Model** | Mention of the names of previous terrorist attackers or violent political leaders (e.g. Breivik, Tarrant, Hitler, etc.) or specific attack references (e.g. Christchurch, Poway, El Paso, Utoya, Halle, etc.), in combination with terms that indicate perceived role model status such as “hero”, “role model”, “saint”, “inspiring”, “inspire”, “inspiration”, “support”, “influenced by” |
|  | **Hopelessness of Alternative Solutions** | “democracy”, “democratic”, “peaceful”, political”, “system”, “politics”, “dialogue”, “passivity” *in combination with* “meaningless”, “weakness”, “failed”, “end”, “vanish”, “man-made”, “jahili”, “all societies existing” |

**Appendix 3: Manual Review Sampling Technique**

By using the grep R function in our NLP analysis, we wanted to capture a wide range of derivations of our selected linguistic markers (e.g., nouns, verbs and adjectives in both singular and plural forms) and thereby minimise the number of false negatives. However, this approach meant that the R-based datasets for each narrative contained a relatively high proportion of false positives—i.e., terms and phrases that were wrongly categorized as a narrative-specific linguistic marker. For instance, a common false positive that was mistaken for a fusion marker by our R code was the use of kinship language by users to speak about their biological family rather than refer to fellow group members as “brothers” and “sisters”. Likewise, the messages the R code identified as instances of out-group dehumanisation (using linguistic markers such as “monkey”, “dog” or “beast”) sometimes contained references to real animals. To address this potential limitation, the datasets of phrases captured by the R code for each narrative category were exported from R and scanned manually for false positives by the lead author. Based on a careful review of all messages, every detected case of a false positive was removed manually from datasets with up to 800 messages.

Due to time constraints, a sampling technique was used for the manual review of large datasets. Whenever a dataset filtered for narrative-specific markers by the R code exceeded 800 messages, the lead author manually reviewed a random sample of 500 messages taken from the respective dataset to determine the percentage of false negatives and applied this percentage to the overall dataset. To ensure that the manually reviewed sample was large enough and the percentage of false positives found in the sample was representative for the entire dataset, a confidence interval was calculated. The confidence interval we used (95% CI < ± 3.5) means that the false positive percentages we computed based on the manual sample review for larger datasets is expected to vary by a maximum margin of error of plus or minus three percentage points at a 95 percent probability. We also tested our datasets for spam activities by conducting a manual review of 100 sample messages posted by the five accounts with the highest number of messages. Based on our review, no spam accounts were detected.

**Appendix 4: Statistical Relevance of Violence Risk Categories**

Table 3. Statistical Relevance of Linguistic Markers found in Terrorist Manifesto Analysis

|  | **Fusion** | **Existential Threat** | **Slurs** | **Demonisation** | **Dehumanisation** | **Conspiracy Belief** | **Inevitable War** | **Justification of Violence** | **Martyrdom Narrative** | **Violent Role Model** | **Hopelessness of Alternative Solutions** | **Calls to Violence** |
| --- | --- | --- | --- | --- | --- | --- | --- | --- | --- | --- | --- | --- |
|  |  |  |  |  |  |  |  |  |  |  |  |  |
| 1. **Violent vs. Non-Violent Significance (p<0,05)** | | | | | | | | | | | | |
| **Significance** | **Yes** | **No** | **Yes** | **Yes** | **Yes** | **No** | **No** | **Yes** | **No** | **No** | **Yes** | **Yes** |
| **n1** | **9** | **9** | **9** | **9** | **9** | **9** | **9** | **9** | **9** | **9** | **9** | **9** |
| **n2** | **6** | **6** | **6** | **6** | **6** | **6** | **6** | **6** | **6** | **6** | **6** | **6** |
| **R1** | **93.5** | **76.5** | **90** | **90** | **95.5** | **87** | **88** | **81** | **88.5** | **84** | **90** | **84** |
| **R2** | **26.5** | **43.5** | **30** | **30** | **24.5** | **33** | **32** | **24** | **31.5** | **36** | **30** | **21** |
| **critical U value** | **10** | **10** | **10** | **10** | **10** | **10** | **10** | **10** | **10** | **10** | **10** | **10** |
| **U1** | **5.5** | **22.5** | **9** | **9** | **3.5** | **12** | **11** | **3** | **10.5** | **15** | **9** | **0** |
| **U2** | **48.5** | **31.5** | **45** | **45** | **50.5** | **42** | **43** | **51** | **43.5** | **39** | **45** | **54** |
| **U value** | **5.5** | **22.5** | **9** | **9** | **3.5** | **12** | **11** | **3** | **10.5** | **15** | **9** | **0** |
| 1. **Extreme vs. Non-Extreme Significance (p<0,05)** | | | | | | | | | | | | |
| **Significance** | **No** | **No** | **No** | **No** | **No** | **No** | **No** | **Yes** | **No** | **No** | **No** | **No** |
| **n1** | **12** | **12** | **12** | **12** | **12** | **12** | **12** | **12** | **12** | **12** | **12** | **12** |
| **n2** | **3** | **3** | **3** | **3** | **3** | **3** | **3** | **3** | **3** | **3** | **3** | **3** |
| **R1** | **109.5** | **89** | **105** | **103** | **109.5** | **108** | **106.5** | **99** | **106.5** | **102** | **105** | **94.5** |
| **R2** | **10.5** | **31** | **15** | **17** | **10.5** | **12** | **13.5** | **6** | **13.5** | **18** | **15** | **10.5** |
| **critical U value** | **4** | **4** | **4** | **4** | **4** | **4** | **4** | **4** | **4** | **4** | **4** | **4** |
| **U1** | **4.5** | **25** | **9** | **11** | **4.5** | **6** | **7.5** | **0** | **7.5** | **12** | **9** | **4.5** |
| **U2** | **31.5** | **11** | **27** | **25** | **31.5** | **30** | **28.5** | **36** | **28.5** | **24** | **27** | **31.5** |
| **U value** | **4.5** | **11** | **9** | **11** | **4.5** | **6** | **7.5** | **0** | **7.5** | **12** | **9** | **4.5** |

**
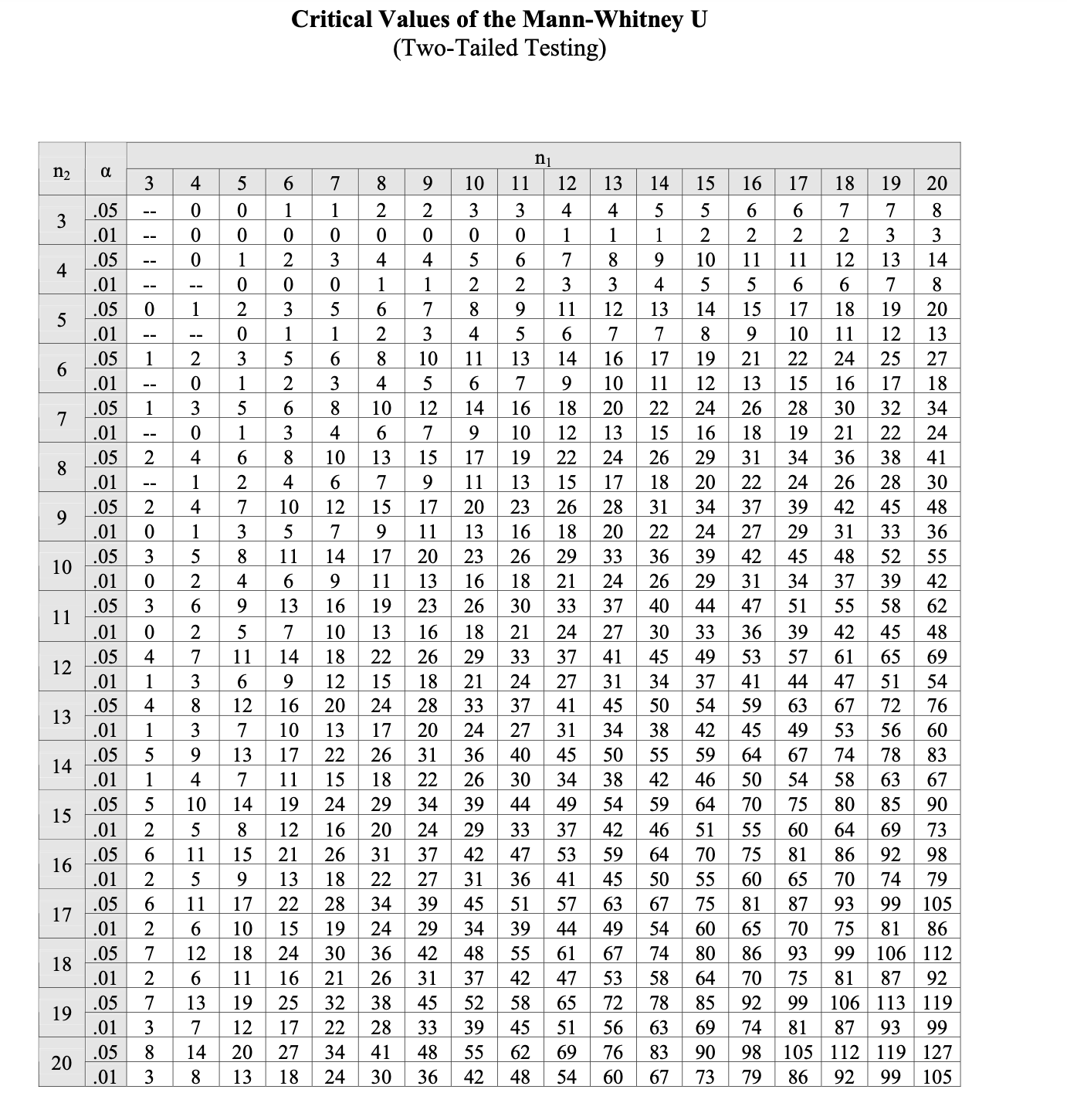
**

1. V.K. Choi, M.J. Gelfand and J.C. Jackson, “The role of entitativity in perpetuating cycles of violence”, *Behavioral and Brain Sciences 41* (2018); B. Lickel, N. Miller, D.M. Stenstrom, T.F. Denson and T. Schmader, “Vicarious Retribution: The Role of Collective Blaming in Intergroup Aggression”, Personality and Social Psychology Review 10, no. 4 (2006): 372-390. [↑](#endnote-ref-1)
2. J.M Berger, “Extremist Construction of Identity: How Escalating Demands for Legitimacy Shape and Define In-Group and Out-Group Dynamics”, ICCT Research Paper, 2017. [↑](#endnote-ref-2)
3. R. Giner-Sorolla, B. Leidner and E. Castano, “Dehumanization, Demonization, and

   Morality Shifting”, in *Extremism and the Psychology of Uncertainty*, ed. M.A. Hogg and D.L. Blaylock (Hoboken, JJ: Wiley-Blackwell, 2011): 165-182. [↑](#endnote-ref-3)
4. Whitehouse, “Dying for the group”. [↑](#endnote-ref-4)
5. Gilad Hirschberger, Tsachi Ein-Dor, Bernard Leidner and Tamar Saguy, “How Is

   Existential Threat Related to Intergroup Conflict? Introducing the Multidimensional Existential

   Threat (MET) Model“, *Frontiers in Psychology 7*, Article 1877 (2016). [↑](#endnote-ref-5)
6. Ali Mashuri and Esti Zaduqisti, “The effect of intergroup threat and social identity salience on the belief in conspiracy theories over terrorism in Indonesia: Collective angst as a mediator”, International Journal of Psychological Research, 8, no. 1 (2015): 24–35. [↑](#endnote-ref-6)
7. Julia Ebner, The Rage: The Vicious Circle of Islamist and Far-Right Extremism (London:

   Bloomsbury Academic, 2017). [↑](#endnote-ref-7)
8. W.R. Louis, C. McGarty, E.F. Thomas, C.E. Amiot and F.M. Moghaddam, “The power of norms to sway fused group members”, *Behavioral And Brain Sciences* *41* (2018): 29-30. [↑](#endnote-ref-8)
9. Louis et al., “The power of norms”. [↑](#endnote-ref-9)
10. Jacob Davey and Julia Ebner, “‘The Great Replacement’: The Violent Consequences

    of Mainstreamed Extremism” (London: Institute for Strategic Dialogue (ISD), 2019) and

    Katie Cohen, Fredrik Johansson, Lisa Kaati and Jonas Clausen Mork, “Detecting Linguistic

    Markers for Radical Violence in Social Media”, *Terrorism and Political Violence 26,* no 1

    (2014): 246-256. [↑](#endnote-ref-10)
11. E. F. Thomas and W.R. Louis, “When will collective action be effective? Violent and

    non-violent protests differentially influence perceptions of legitimacy and efficacy amongst

    supporters“, *Personality and Social Psychology Bulletin 40* (2014): 263–76. [↑](#endnote-ref-11)
12. E.A. Bäck, H. Bäck, M.G. Sendén and S. Sikström, “From I to We: Group Formation and

    Linguistic Adaption in an Online Xenophobic Forum“, *Journal of Social and Political*

    *Psychology 6,* no. 1 (2018): 76-91. [↑](#endnote-ref-12)
13. Whitehouse and Lanman, “The Ties That Bind Us”. [↑](#endnote-ref-13)
14. Björn Technau, “Going beyond hate speech: The pragmatics of ethnic slur terms”, Lodz *Papers in Pragmatics 14*, no. 1 (2018): 25-43. [↑](#endnote-ref-14)
15. L. Anderson, and E. Lepore, “Slurring Words”, *Nous 47* (2013): 25–48. [↑](#endnote-ref-15)
16. Robin Jeshion, “Slurs and Stereotypes“, *Analytic Philosophy 54*, no 3 (2013): 314-329. [↑](#endnote-ref-16)
17. Nahi Alon and Haim Omer, *The Psychology of Demonization: Promoting Acceptance and*

    *Reducing Conflict* (London: Routledge, 2005). [↑](#endnote-ref-17)
18. Elihu D. Richter, Dror Kris Markus and Casey Tait, “Incitement, genocide, genocidal terror,

    and the upstream role of indoctrination: can epidemiologic models predict and prevent?“, *Public*

    *Health Review 39: 30* (2018). [↑](#endnote-ref-18)
19. Monika Schwarz-Friesel. “Using Language as a Weapon: Verbal Manifestations of

    Contemporary Anti-Semitism“ In *The Exercise of Power in Communication* , ed. Rainer

    Schulze and Hanna Pishwa (London: Palgrave Macmillan, 2015), 161-183. [↑](#endnote-ref-19)
20. Jeroen Vaes, Jacques-Philippe Leyens, Maria Paola Paladino and Mariana Pires Miranda.

    “We are human, they are not: Driving forces behind outgroup dehumanisation and the

    humanisation of the ingroup,“ *European Review of Social Psychology 2*, no. 1 (2012): 64-106. [↑](#endnote-ref-20)
21. G.T. Viki, L. Winchester, L. Titshall and T. Chisango, ”Beyond secondary emotions:

    The infra-humanisation of groups using human-related and animal-related words”, *Social*

    *Cognition 24* (2006): 753–775. [↑](#endnote-ref-21)
22. Cf. Hirschberger et al, “How is Existential Threat Related to Intergroup Conflict?”. [↑](#endnote-ref-22)
23. Cynthia Miller-Idriss, “White Supremacist Extremism and the Far Right in the U.S.“

    (Farmington Hills: Gale Primary Sources/Cengage, 2021). [↑](#endnote-ref-23)
24. J.M., Bale, “Political paranoia v. political realism: On distinguishing between bogus

    conspiracy theories and genuine conspiratorial politics“, *Patterns of Prejudice 41* (2007):45–60. [↑](#endnote-ref-24)
25. C. Klein,, P. Clutton and A.G. Dunn, “Pathways to conspiracy: The social and linguistic

    precursors of involvement in Reddit’s conspiracy theory forum“, *PLoS ONE 14, no. 11 (*2019). [↑](#endnote-ref-25)
26. Matthew Kriner, Meghan Conroy and Yasmine Ashwal, “Understanding Accelerationist

    Narratives: ‘There Is No Political Solution’” (London: GNET Report, Kings College, 2021). [↑](#endnote-ref-26)
27. Winnifred R. Louis, Donald M. Taylor and Rebecca L. Douglas, “Normative Influence

    and Rational Conflict Decisions: Group Norms and Cost-Benefit Analyses for Intergroup Behavior“, *Group Processes & Intergroup Relations 8,* no. 4 (2005): 355-374 and Dario Spini, Guy Elcheroth and Rachel Fasel, “The Impact of Group Norms and Generalization of Risks across Groups on Judgments of War Behavior”, *Political Psychology 29*, no. 6 (2008): 920-941. [↑](#endnote-ref-27)
28. Mohammed M. Hafez, “Martyrdom Mythology in Iraq: How Jihadists Frame Suicide

    Terrorism in Videos and Biographies“, *Terrorism and Political Violence 19,* no. 1 (2007): 95-115 and Thomas Fraise, “Shades of jihad: Variation of military ethics between ISIS and alQaeda*”,* (Paris: Sciences Po Kuwait Program, Research Paper, 2017). [↑](#endnote-ref-28)
29. Cf. Rola el-Husseini, “Resistance, Jihad, and Martyrdom in Contemporary Lebanese Shi'a

    Discours“, *Middle East Journal 62, no. 3 (*2008): 399-414; Hafez, ”Martyrdom Mythology in Iraq” and Amélie Blom, “Do Jihadist ‘Martyrs’ really want to die? An emic approach to self-sacrificial radicalization in Pakistan”, *Revue française de science politique 61* (2011): 867-891. [↑](#endnote-ref-29)
30. Richter, Markus and Tait, “Incitement, Genocide, Genocidal Terror”. [↑](#endnote-ref-30)
31. Davey and Ebner, “’The Great Replacement’” and Cohen et al., “Detecting Linguistic Markers for Radical Violence”. [↑](#endnote-ref-31)
32. Thomas and Louis, “When will collective action be effective?“ and R. Spears “Group

    rationale, collective sense: Beyond intergroup bias“, *British Journal of Social Psychology 49*

    (2010): 1-20. [↑](#endnote-ref-32)
33. Cohen et al., “Detecting Linguistic Markers for Radical Violence”. [↑](#endnote-ref-33)
